# Low Plasma Leptin and High Soluble Leptin Receptor Levels Are Associated With Mild Cognitive Impairment in Type 2 Diabetic Patients

**DOI:** 10.3389/fnagi.2018.00132

**Published:** 2018-05-15

**Authors:** Han Yin, Sai Tian, Rong Huang, Rongrong Cai, Dan Guo, Hongyan Lin, Jiaqi Wang, Shaohua Wang

**Affiliations:** Department of Endocrinology, The Affiliated ZhongDa Hospital of Southeast University, Nanjing, China

**Keywords:** leptin, visceral obesity, waist-to-hip ration, mild cognitive impairment, type 2 diabetes

## Abstract

**Background:** Metabolic disturbances, such as hyperglycemia, hyperlipidemia, and obesity, are thought to be risk factors for Alzheimer's disease.

**Objective:** This study aimed to investigate whether adipokine leptin and soluble leptin receptor (sOBR) levels are correlated with mild cognitive impairment (MCI) and metabolic status of the patients of type 2 diabetes mellitus (T2DM).

**Methods:** Total of 169 T2DM patients were recruited and divided according to the Montreal Cognitive Assessment (MoCA) score. Their cognition and metabolic parameters were extensively assessed. Plasma leptin and sOBR levels were determined by RIA and ELISA, respectively. Free leptin index (FLI) was calculated.

**Results:** Of 124 enrolled T2DM patients, 61 were diagnosed with MCI (MoCA < 26). In MCI group and female subgroup, leptin levels and FLI were significantly lower and sOBR concentrations were higher when compared with their respective health cognition controls (all *p* < 0.05). Leptin levels and FLI were positively associated with the most cognitive domains and their Waist-to-Hip Ratio (WHR), a traditional index of central obesity. Leptin, sOBR, and WHR were independent variables of MCI in all individuals (all *p* < 0.05). For the females, MoCA was also positively correlated with leptin (β = 0.893, *p* < 0.001) and negatively correlated with WHR (β = −0.363, *p* = 0.014). Leptin was positively correlated only with WHR (β = 0.441, *p* = 0.025).

**Conclusions:** This study showed that a high level of leptin and low level of sOBR are associated with the improved cognitive function in T2DM patients and more significantly in female individuals, while WHR, as an indicator of the visceral obesity, contributes to cognitive deficits.

**Trial registration:** Advanced Glycation End Products Induced Cognitive Impairment in Diabetes: BDNF Signal Meditated Hippocampal Neurogenesis ChiCTR-OCC-15006060; http://www.chictr.org.cn/showproj.aspx?proj=10536.

## Introduction

With the development of society and the acceleration of population aging, diabetes mellitus (DM) is becoming one of the most prevalent chronic diseases affecting more than 250 million people around the world (Luchsinger, 2010). People with type 2 diabetes mellitus (T2DM) are at a high risk for developing cognitive dysfunction and dementia (e.g., Alzheimer's disease), which has become a major public health concern (Munshi, 2017; Simó et al., 2017).

There is increasing evidence to indicate that alterations in body weight and systemic metabolism (e.g., obesity, underweight, hyperglycemia, hyperlipidemia etc.) play an important role in mild cognitive impairment (MCI), a transitional stage between normal cognition and Alzheimer's disease (AD) (Emmerzaal et al., 2015; Kim and Feldman, 2015). Several studies showed that obesity (high BMI) or central obesity had an increased risk of developing cognition decline and AD (Luchsinger et al., 2012; Zeki Al Hazzouri et al., 2012; Arnoldussen et al., 2014). The possible pathogenesis of which include inflammation, insulin resistant and adipokines dysfunction (e.g., leptin) (Lee, 2011; Kiliaan et al., 2014).

Leptin, an adipocyte-derived hormone, circulate in a free and a bound form to its receptors. In human blood, the primary binding protein for leptin is the soluble leptin receptor (sOB-R), acting as a buffer to modulate the bioavailability of leptin. Through the receptors expressed within the hypothalamus, the cortex and hippocampus, leptin not only serve as a regulator of body weight, fat storage, and systemic metabolism by modulating energy intake, glucose utilization and insulin sensitivity but also exert effects in cognitive function (Coppari and Bjørbæk, 2012; Knights et al., 2014). Recent evidence from cellular, animal and human studies show the effects of leptin on neuroprotection which involves several mechanisms, including the regulation of beta amyloid synthesis, clearance of tau protein phosphorylation, protection against oxidative stress and attenuation of apoptotic cell death et al. (Irving and Harvey, 2014; Farr et al., 2015). In a rodent model, hippocampus infusion of leptin by viral vector results in decreased neurodegeneration and increased neuronal precursor proliferation (Pérez-González et al., 2011). A few human observational studies also reported a protective effect of leptin on cognitive decline and dementia in nonobese individuals (Holden et al., 2009; Lieb et al., 2009; McGuire and Ishii, 2016). Thus, an intriguing link between body weight status and brain function was suggested through adipocyte hormone leptin.

Furthermore, although metabolic disorder especially obesity and hyperglycemia was found to increase the risk of mild cognitive impairment and AD, and a strong association between leptin level and cognitive function was previously demonstrated, to the best of our knowledge, little is known about the link between leptin and cognitive function and metabolic disturbances in T2DM. Currently, we investigate the association of leptin, sOBR and free leptin index (FLI, a marker of leptin activity) with mild cognitive impairment stratified by body weight status (normal, overweight and obese by BMI) in diabetic patients to explore whether leptin, sOBR, and obesity may be better predictive markers and therapeutic target for diabetic patients with MCI.

## Materials and methods

### Clinical subjects and assessment

This study was conducted in the Endocrinology department of the Affiliated ZhongDa Hospital of Southeast University. The Affiliated ZhongDa Hospital of Southeast University Research Ethics Committee approved the study. Each participant provided a written informed consent before participation in the study.

We recruited a total of 169 hospitalized patients aged 40–80 years who met the diagnostic criteria of T2DM between August 2015 and March 2016. One hundred and twenty-four patients remained for further analysis (45 patients failed to complete the study due to economic reasons).Of the 124 individuals, there were 61 diabetic patients with MCI (Montreal Cognitive Assessment [MoCA] < 26; 31 men and 30 women) and 63 age-matched diabetic patients with healthy cognition as control (MoCA ≥ 26; 34 men and 29 women). All of the MCI patients satisfied the following diagnostic criteria for MCI proposed by the MCI Working Group of the European Consortium on Alzheimer's disease in 2006: (1) cognitive complaints coming from patients or their families; (2) reporting a decline in cognitive functioning relative to that of the past year by the patient or informant (CDR score of 0.5); (3) cognitive disorders as evidenced by clinical evaluation (impairment in memory or some other cognitive domain); (4) absence of major repercussions on daily life (ADL score < 26); and (5) absence of dementia (based on the DSM-IV criteria). The following exclusion criteria were considered in the study: history of known stroke (Hachinski ischemic score ≥ 4), head injury, alcoholism, epilepsy, Parkinson's disease, major depression (excluded by Self-Rating Depression Scale) or other neurological or psychiatric illnesses (e.g., anxiety, substance dependence and personality disorder) excluded by clinical assessment and case history, or significant medical illnesses (e.g., cancer, anemia, and thyroid dysfunction) and use of medication that could interfere with cognitive testing (including anti-Parkinson's disease drugs; central B-blockers; nerve sedatives; benzodiazepines; narcotic analgesics; barbiturates; sedative medicines or short-acting anxiolytic used more than twice a week; or any drug recently approved for AD).

### Clinical data

Demographic characteristics, including age, gender, education level, occupation, physical measurement (including blood pressure, height, weight, WC [waist circumferences], HC [hip circumference]. WHR, calculated as WC/HC. BMI, calculated as weight/height^2^ [Kg/m^2^]. Normal, overweight and obese are defined as BMI between 18.5 and 23.9 kg/m^2^, 24 and 28 kg/m^2^, and BMI ≥ 28 kg/m^2^, respectively according to the Chinese WGOC definition), a detailed medical history of diabetic patients: diabetes duration, current treatment, co-morbid diseased (including coronary heart disease, fatty liver, hypertension, cerebral infarction and other), smoking, drinking, ethnicity, and contact information were carefully collected. Blood samples were obtained to determine fasting blood-glucose (FBG), fasting C-peptide (FCP), 2 h postprandial glucose (2 hPG), glycosylated hemoglobin (HbA1c), homeostasis model of assessment for insulin resistance (HOMA-IR), serum creatinine, serum uric acid, anti-human antibody apolipoprotein A1 (ApoA1), anti-human antibody apolipoprotein B (ApoB), TC (total cholesterol), TG, low density lipoprotein (LDL-C), and high density lipoprotein (HDL-C). Urine samples were obtained for microalbuminuria, urine creatinine. Largest amplitude of glycemic excursions (LAGE) was recorded by CGMS. The central laboratory of the Affiliated Zhongda Hospital of Southeast University implements internal and external quality control procedures directed by the Chinese Laboratory Quality Control. ABI (Ankle-Brachial Index), calculated as Ankle pressure/ highest arm pressure. The lowest value of the ABI was used in the analyses. Tcpo2 was determined by Clark-electrode based tcpo2 monitoring device attached to the dorsum skin of the foot.

### Neuropsychological tests

A battery of neuropsychological tests, including MoCA, Mini Mental State Exam (MMSE), Rey-Osterreith Complex Figure Test (CFT), Auditory Verbal Learning Test (AVLT), Digit Span Test (DST), Trail Making Test-A and B (TMT-A and TMT-B), Similarities Test (ST), Clock Drawing Test (CDT), Logic memory test (LMT), Stroop effect test and Verbal Fluency Test (VFT) were performed to evaluate the function of verbal and visual information, attention, semantic memory, executive function, psychomotor speed, and visuospatial skills. Hachinski ischemic core, clinical dementia rating (CDR), activity of daily living scale (ADL), and self-rating depression scale (SDS) were also included. The experienced neurologist and all of the subjects were blinded to the study design. None of the participants displayed audiovisual or motor coordination deficits affecting the test.

### Determination of plasma leptin and soluble leptin receptor

Blood samples were collected by venipuncture and centrifuged at 4°C. Plasma samples were stored at −80°C prior to analysis. The plasma level of leptin and leptin receptor was measured on the same day to reduce assay variance. The plasma leptin and soluble leptin receptor concentrations were determined by RIA method (Linco Research Inc.) and enzyme-linked immunosorbent assay (ELISA) kits (BioVendor) according to the manufacturer's instructions, respectively. For leptin and leptin receptor, intra and inter-assay coefficients of variation were <10%, and the sensitivities were 0.5 and 0.8 ng/ml, respectively. Free leptin index as a marker of leptin activity was calculated according to the following formula: plasma leptin concentration (ng/ml)/plasma soluble leptin receptor concentration (ng/ml) × 100.

### Statistical analysis

Data was conducted by using SPSS-19 (SPSS Inc., Chicago, IL, USA). Quantitative variables were expressed as means ± standard deviations (SDs) or median (interquartile range) as appropriate, and qualitative variables as percentages. Student's *t*-test and analysis of variance (ANOVA) were applied to compare normally distributed variables; nonparametric Mann-Whitney *U* tests were employed for asymmetrically distributed variables. Chi-square (χ^2^) test was conducted to compare qualitative variables. The correlations between plasma Leptin, sOBR, and FLI with clinical factors were analyzed by using partial correlation analysis. Multivariable regression analysis and forward stepwise binary logistic regression analysis were performed to investigate the relationship between the cognitive measures and demographic factors. All statistical tests were two-sided. A *p*-value of less than 0.05 was defined as statistically significant.

## Results

### Demographic and clinical characteristics

Demographic characteristics, use of medications, clinical data, and neuropsychological test scores are presented in Table 1.

**Table 1:** Demographic, clinical, and cognitive characteristics in MCI group compared with the control group.

**Characteristic**	**MCI group (*****n*** = **61)**			**Con group (*****n*** = **63)**		
	**Total (*n* = 61)**	**Men (*n* = 31)**	**Women (*n* = 30)**	**Total (*n* = 63)**	**Men (*n* = 34)**	**Women (*n* = 29)**
Age (y)	60.70 ± 7.01	59.97 ± 7.80	61.47 ± 6.12	58.27 ± 8.87	56.71 ± 8.48	60.10 ± 9.10
Female (%)	30 (49.2)			29 (46)		
Diabetes duration (y)	11.44 ± 5.57	11.52 ± 6.31	11.37 ± 4.80	10.34 ± 5.72	9.10 ± 5.23	11.83 ± 5.75
Insulin use, *n* (%)	35 (57.38)	18 (58.10)	17 (56.70)	35 (55.56)	17 (50.00)	18 (62.07)
Metformin use, *n* (%)	36 (59.02)	20 (64.52)	16 (53.33)	34 (53.97)	15 (44.12)	19 (65.52)
Hypertension, *n* (%)	39 (63.93)	16 (51.61)	23 (76.67)	32 (50.79)	16 (47.06)	16 (55.17)
Hypertention duration (y)	5.00 (0.00–12.75)	1.00 (0.00–12.00)	8.00 (0.75–14.25)	3.00 (0.00–10.00)	0.00 (0.00–10.00)	3.00 (0.00–10.00)
Education levels (y)	10.20 ± 2.83	10.74 ± 3.14	9.63 ± 2.39	11.13 ± 2.52	11.76 ± 2.51	10.38 ± 2.35
Fatty liver, *n* (%)	30 (49.20)	14 (45.16)	16 (53.33)	24 (38.10)	13 (38.24)	11 (37.93)
Smoking, *n* (%)	20 (32.80)	19 (61.29)	1 (3.33)	21 (33.33)	19 (55.88)	2 (6.89)
Drinking, *n* (%)	12 (19.70)	10 (32.26)	2 (6.67)	12 (19.00)	11 (32.35)	1 (3.45)
SBP (mmHg)	136.38 ± 19.42	131.52 ± 16.86	141.40 ± 20.86	135.38 ± 14.83	134.09 ± 14.84	136.90 ± 14.94
DBP (mmHg)	81.25 ± 11.40	80.68 ± 10.16	81.3 ± 12.38	80.03 ± 11.24	81.88 ± 10.92	77.86 ± 11.41
BMI (kg/m2)	24.78 (23.03–27.24)	24.21 (22.38–26.09)	25.15 (23.96–29.26)	24.22 (22.76–27.18)	23.97 (21.38–24.91)	25.89 (23.49–27.73)
Normal weight (%)	24 (39.34)	15 (48.39)	7 (23.33)	27 (42.86)	18 (52.94)	9 (31.03)
Overweight (%)	25 (40.98)	13 (41.94)	14 (46.67)	24 (38.09)	10 (29.41)	14 (48.28)
Obese (%)	12 (19.67)	3 (9.68)	9 (30.00)	12 (19.05)	6 (17.65)	6 (20.69)
WC (cm)	91.40 ± 8.03	91.04 ± 6.92	91.77 ± 9.14	88.89 ± 9.52	88.24 ± 9.04	89.64 ± 10.16
WHR	0.952 ± 0.06	0.953 ± 0.039	0.95 ± 0.071	0.943 ± 0.058	0.950 ± 0.059	0.935 ± 0.058
FPG (mmol/L)	8.44 ± 2.84	8.49 ± 3.11	8.28 ± 2.49	7.90 ± 2.80	8.46 ± 3.05	7.24 ± 2.36
2 h PG (mmol/L)	15.28 ± 3.75	15.63 ± 3.77	14.93 ± 3.77	14.66 ± 4.48	14.86 ± 4.67	14.43 ± 4.31
LAGE	7.93 ± 3.31	8.26 ± 3.72	7.59 ± 2.84	7.50 ± 3.79	7.56 ± 4.00	7.43 ± 3.59
HbA1c (%)	9.00 (8.10–11.40)	9.50 (8.10–11.90)	8.85 (8.07–10.90)	8.80 (7.40–10.20)	9.3 (7.13–10.70)	8.00 (7.40–9.90)
FCP	1.23 (0.69–1.69)	1.04 (0.63–1.70)	1.36 (0.77–1.71)	1.02 (0.589–1.71)	1.07 (0.58–1.73)	1.00 (0.57–1.72)
HOMA-IR	0.46 (0.24–0.60)	0.42 (0.22–0.62)	0.50 (0.33–0.60)	0.33 (0.19–0.55)	0.32 (0.17–0.61)	0.31 (0.19–0.49)*
TC (mmol/L)	4.65 ± 1.05	4.42 ± 0.73	4.88 ± 1.27	4.58 ± 1.15	4.52 ± 1.06	4.65 ± 1.26
TG (mmol/L)	1.52 (0.93–2.27)	1.48 (0.92–2.37)	1.65 (0.94–2.25)	1.47 (0.94–2.14)	1.49 (1.08–2.22)	1.37 (0.94–1.93)
LDL (mmol/L)	2.91 ± 0.85	2.76 ± 0.72	3.07 ± 0.94	2.87 ± 0.857	2.82 ± 0.823	2.93 ± 0.91
HDL (mmol/L)	1.09 (0.95–1.35)	1.06 (0.9–1.26)	1.14 (1.00–1.43)	1.16 (0.92–1.35)	1.04 (0.88–1.35)	1.19 (1.05–1.42)
APOA1 (g/L)	1.03 (0.87–1.27)	0.94 (0.84–1.25)	1.11 (0.96–1.33)	1.07 (0.83–1.22)	0.95 (0.785–1.233)	1.12 (0.99–1.23)
APOB (mmol/L)	0.82 (0.68–0.92)	0.77 (0.65–0.87)	0.85 (0.75–1.12)	0.82 (0.68–0.94)	0.81 (0.658–0.943)	0.82 (0.72–0.95)
LPa (mmol/L)	211.0 (94.50–334.8)	153.5 (75.80–273.5)	250.0 (112.5–452.3)	226.0 (108.0–352.0)	191.5 (107.3–345.3)	231.0 (93.50–405.0)
ABI	1.10 (1.05–1.15)	1.14 (1.06–1.17)	1.09 (1.04–1.13)	1.13 (1.06–1.17)	1.14 (1.07–1.18)	1.10 (1.04–1.16)
Tcpo2 (mmHg)	52.74 ± 12.09	54.35 ± 14.19	51.07 ± 9.41	55.67 ± 12.07	58.47 ± 12.20	52.38 ± 11.25
Leptin (ng/ml)	3.49 ± 1.81	1.93 ± 0.14	5.11 ± 0.17	5.33 ± 3.73*a	2.16 ± 0.15	9.05 ± 0.34*a2
SOBR (ng/ml)	50.77 ± 16.17	42.15 ± 2.38	59.67 ± 2.57	40.11 ± 14.07*a	34.81 ± 1.59	46.34 ± 3.01*a2
FLI	7.26 ± 3.67	5.52 ± 3.56	9.07 ± 2.86	14.01 ± 9.49*b	6.64 ± 2.96	22.65 ± 11.79*b2
**COGNITION TEST LEVELS**						
LMT	6.13 ± 4.06	7.71 ± 3.87	4.50 ± 3.65	9.81 ± 4.19 a ^**^a	9.74 ± 4.41^*^a1	9.90 ± 4.00^**^a2
AVLT–delayed recall	4.44 ± 2.46	5.00 ± 2.57	3.87 ± 2.24	6.24 ± 2.18 a ^**^a	6.03 ± 2.26	6.48 ± 2.08^**^a2
AVLT immediate recall	15.34 ± 5.13	15.65 ± 5.24	15.03 ± 5.09	18.95 ± 4.26 a ^**^a	18.15 ± 3.5^*^a1	19.9 ± 4.89^**^a2
VFT	13.95 ± 3.05	14.71 ± 2.51	13.17 ± 3.39	16.81 ± 3.74 a ^**^a	17.06 ± 3.96^*^a1	16.52 ± 3.49^**^a2
DST	10.46 ± 2.05	10.94 ± 2.02	9.97 ± 1.99	12.05 ± 1.90 a ^**^a	12.21 ± 1.74^*^a1	11.86 ± 2.08^**^a2
MOCA	22.00 (19.50–24.00)	24 (21.00–24.00)	20.50 (17.00–23.00)	27.00 (26.00–27.00)^**^b	27.00 (26.00–27.25)^**^b1	27.00 (26.00–27.50)^**^b2
MMSE	26.00 (25.00–28.00)	27.00 (26.00–28.00)	25.00 (23.00–27.00)	29.00 (28.00–30.00)^**^b	29.00 (28.00–30.00)^**^b1	29.00 (28.00–30.00)^**^b2
CDT	3.00 (2.00–4.00)	3.00 (2.00–3.00)	3.00 (2.00–4.00)	4.00 (3.00–4.00)^*^a	4.00 (3.00–4.00)	4.00 (3.00–4.00)^*^b2
TMTA	67.0 (54.00–92.50)	64.00 (51.00–77.00)	72.50 (55.00–96.25)	55.00 (45.00–67.00)^**^b	49.50 (41.75–65.00)^*^b1	56.00 (48.50–72.00)^*^b2
TMTB	179.0 (150.0–244.0)	173.0 (141.0–216.0)	195.5 (166.0–269.5)	138.0 (106.0–179.0)^**^b	132.0 (95.00–181.8)^*^b1	148.0 (113.5–180.5)^**^b2
Stroop card A (time)	33.00 (28.50–38.50)	32.00 (29.00–40.00)	35.50 (25.00–38.00)	28.00 (23.00–36.00)^*^b	28.00 (23.00–34.25)^*^b1	28.00 (23.50–37.50)
Stroop card A (number)	50.00 (49.50–50.00)	50.00 (49.00–50.00)	50.00 (49.75–50.00)	50.00 (50.00–50.00)	50.00 (50.00–50.00)^*^b1	50.00 (49.50–50.00)
Stroop card B (time)	59.00 (48.00–64.50)	58.00 (48.00–65.00)	59.50 (44.00–62.75)	42.00 (36.00–50.00)^**^b	41.50 (35.75–47.00)^**^b1	44.00 (36.50–56.00)^*^
Stroop card B (number)	47.00 (46.00–50.00)	48.00 (46.00–50.00)	47.00 (45.75–49.25)	50.00 (49.00–50.00)^**^b	50.00 (50.00–50.00)^**^b1	50.00 (48.00–50.00)^*^b2
Stroop card C (time)	112.0 (84.50–124.5)	112.0 (84.00–125.0)	112.5 (83.25–123.0)	82.00 (72.00–95.00)^**^b	82.50 (70.75–96.00)^*^b1	82.00 (72.00–93.00)^**^b2
Stroop card C (number)	44.00 (42.00–47.00)	44.00 (42.00–47.00)	44.50 (42.00–46.50)	48.00 (46.00–50.00)^**^b	48.00 (45.75–50.00)^*^b1	48.00 (45.50–50.00)^**^b2

Data are presented as n (%), mean ± SD, or median (interquartile range) as appropriate. a, Student's t-test for comparison of normally distributed quantitative variables between MCI group and control group. a1, Student's t-test for comparison of normally distributed quantitative variables between MCI men group and control men group. a2, Student's t-test for comparison of normally distributed quantitative variables between MCI women group and control women group. b, Mann–Whitney U-test for comparison of asymmetrically distributed quantitative variables between MCI group and control group. b1, Mann–Whitney U-test for comparison of asymmetrically distributed quantitative variables between MCI men group and control men group. b2, Mann–Whitney U-test for comparison of asymmetrically distributed quantitative variables between MCI women group and control women group. MCI, mild cognitive impairment; BMI, body mass index; ABI, ankle brachial index; ACR, urine albumin and creatinine ratio; FLI, Free leptin index; sOBR, soluble leptin receptor; FBG, fasting blood-glucose; FCP, fasting C-peptide; LAGE, large maximum amplitude of glycemic excursion; HOMA-IR, homeostasis model of assessment for insulin resistance; HbA1c, glycosylated hemoglobin; ApoA1, apolipoprotein A1; TG, triglyceride; TC, total cholesterol; LDL, low density lipoprotein; HDL, high density lipoprotein; WHR, Waist-to-Hip Ratio; WC, Waist circumference; MOCA, Montreal Cognitive Assessment; MMSE, Mini-mental State Examination; DST, Digit Span Test; VFT, Verbal Fluency Test; TMTA, Trail Making Test-A; TMTB, Trail Making Test-B;AVLT, Auditory Verbal Learning Test; LMT, Logical Memory Test; CDT, Clock Drawing Test; ST, Similarities test;

* *p < 0.05*,

** *p < 0.01 compared with men or women in MCI group*.

The MCI group and control group are well-matched for age, sex, education level, diabetes duration and history of smoking and drinking.

The neuropsychological test scores were significantly lower in the MCI group than that in the control group (all *p* < 0.05).

Further stratification study showed HOMA-IR value was significantly higher in MCI group than those with normal cognitive function in women (*p* = 0.005).

The MCI group exhibited significantly lower levels of leptin and FLI, and a higher level of sOBR compared with the control group. A similar tendency was observed in MCI patients by gender analysis, while the significant difference was observed only in female patients (*p* < 0.05).

### Correlations of plasma leptin, SoBR, and FLI with clinical indicators

In this study, the correlation among the leptin, sOBR, and FLI and clinical parameters was assessed by partial correlation analysis. Among both 124 diabetic patients and 59 diabetic women, after adjusting age, sex, and education levels, a significant positive correlation was seen between leptin levels & FLI and MOCA, MMSE, meanwhile, sOBR negatively correlated with MOCA, MMSE (Table 2). Besides, leptin levels and FLI were found to correlate with WHR and WC positively.

**Table 2:** Correlations of plasma leptin, sOBR, and FLI with clinical and cognitive indicators in all patients and diabetic women.

	**leptin**				**FLI**				**sOBR**			
	**Total (*****n*** = **124)**		**Women (*****n*** = **59)**		**Total (*****n*** = **124)**		**Women (*****n*** = **59)**		**Total (*****n*** = **124)**		**Women (*****n*** = **59)**	
	***r***	*p*-value^*^	***r***	***p*-value**	***r***	***p*-value**	***r***	***p*-value**	***r***	***p*-value**	***r***	***p*-value**
WHR	0.471	0.002	0.407	0.002	0.274	0.002	0.22	0.101	−0.035	0.702	0.067	0.622
WC	0.294	0.001	0.272	0.041	0.211	0.021	0.192	0.152	−0.044	0.631	0.008	0.95
MOCA	0.533	0.000	0.629	0.000	0.423	0.000	0.49	0.000	−0.327	0.000	−0.372	0.004
MMSE	0.444	0.000	0.526	0.000	0.356	0.000	0.42	0.000	−0.264	0.004	−0.331	0.012
DST	0.180	0.049	0.242	0.07	0.131	0.153	0.151	0.261	−0.117	0.202	−0.077	0.567
VFT	0.309	0.001	0.444	0.001	0.238	0.009	0.358	0.009	−0.135	0.143	−0.183	0.172
TMTA	−0.24	0.008	−0.297	0.025	−0.197	0.031	−0.25	0.059	0.165	0.071	0.224	0.094
TMTB	−0.3	0.001	−0.358	0.006	−0.233	0.010	−0.27	0.046	0.181	0.047	0.202	0.131
LMT	0.324	0.000	0.48	0.000	0.283	0.002	0.435	0.001	−0.124	0.176	−0.298	0.024

*WHR, Waist-to-Hip Ratio; WC, Waist circumference; MOCA, Montreal Cognitive Assessment; MMSE, Mini-mental State Examination; DST, Digit Span Test; VFT, Verbal Fluency Test; TMTA, Trail Making Test-A; TMTB, Trail Making Test-B;AVLT, Auditory Verbal Learning Test; LMT, Logical Memory Test*.

* *partial correlation analysis adjusted for age, sex, and education levels*.

Leptin and FLI related to more neuropsychological test scores than the receptor did. For example, among the total 124 diabetic patients, leptin and FLI showed significant association with TMTA, TMTB, AVLT delay and LMT, but the association was only found between TMTB with the receptor. In the female patient, leptin and FLI showed significant association with TMTB, AVLT delay, LMT, Stroop card B (number) and VFT, and the receptor was associated with LMT and Stroop C (number) (Table 2).

### Binary regression analysis for the whole group

We constructed a binary regression model to clarify independent factors that are associated with the prevalence of MCI in 124 patients. All the independent variables in Table 1 were entered in the models. WHR (*B* = 0.546, *p* = 0.011), leptin (*B* = −5.874, *p* = 0.002) and sOBR (*B* = 0.17, *p* = 0.021) were finally introduced to this model (Table 3), which indicated that levels of leptin, sOBR and WHR were independent determinants of MCI in diabetic patients.

**Table 3:** Independent risk factors of MCI in two groups.

	***B***	***p*-value**	**OR**	**95% CI**
WHR	0.546	0.011	1.727	1.135–2.628
Leptin	−5.874	0.002	0.003	0.000–0.119
sOBR	0.17	0.002	1.185	1.062–1.323

*WHR, Waist-to-Hip Ratio; sOBR, soluble leptin receptor*.

### Multivariable regression analysis in diabetic women

Multivariable regression analysis showed MOCA as the dependent variable and clinical parameters, leptin levels, FLI and sOBR as independent variables exhibited in this model (*R*^2^ = 0.699; *p* = 0.001), MOCA significantly positively correlated only with leptin (β = 0.893, *p* < 0.001) and negatively correlated with WHR (β = −0.363, *p* = 0.014) in diabetic women (Table 4).The similar result was found in another multivariable regression analysis with MMSE as the dependent variable (Table 4). Interestingly, when leptin was identified as the dependent variable, which was significantly positively correlated only with WHR (β = 0.441, *p* = 0.025, Table 5).

**Table 4:** Multiple regression analyses exploring the relationship between clinical indicators and MOCA and MMSE in diabetic women.

	***B***	***SE***	**β**	***t***	**Sig**.	**95%CI**
**MOCA**						
Constant	39.14	7.789				
WHR	−25.182	9.851	−0.363	−2.556	0.014	−45.02~−5.34
Leptin	1.641	0.285	0.893	5.761	0.000	1.07~2.22
**MMSE**						
Constant	37.62	7.212				
WHR	−18.77	7.79	−0.445	−2.409	0.021	−34.57~−2.97
Leptin	0.837	0.226	0.749	3.701	0.001	0.378~1.296

*WHR, Waist-to-Hip Ratio*.

**Table 5:** Multiple regression analyses exploring the relationship between leptin and other clinical indicators in diabetic women.

	***B***	***SE***	**β**	***t***	**Sig**.	**95%CI**
**LEPTIN**						
Constant	−3.604	6.231				
WHR	16.65	7.205	0.441	2.311	0.025	2.15~ 31.15

*WHR, Waist-to-Hip Ratio*.

## Discussion

In the present study, we suggested lower plasma leptin level and FLI and a higher level of sOBR in T2DM with MCI compared with the controls, MoCA positively correlated with leptin and negatively correlated with WHR, especially in females. Low leptin, high sOBR level, and high WHR instead of conventional variables were finally determined as independent risk factors of MCI.

This is consistent with the previous findings reported by Holden et al. who suggested higher leptin concentration and lower sOBR were correlated with a less expressed cognitive decline in the elderly in a prospective, longitudinal cohort study (Holden et al., 2009). The data from Khemka also confirmed that lower level of leptin was found in subjects with both AD and MCI compared with the controls (Khemka et al., 2014). Moreover, rat models showed leptin treatment improved memory and learning long-term performance potentiation and modulated hippocampal synaptic plasticity (Oomura et al., 2006). Another study of leptin replacement treatment in genetically leptin-deficient adults also showed positive changes in gray matter volume (Baicy et al., 2007). However, Lieb et al. found the neuroprotective of leptin was only observed in nonobese individuals (Lieb et al., 2009). Teunissen and colleagues also found no correlations between leptin levels and MMSE, the annual change in MMSE or hippocampal atrophy in demented subjects during follow-up (Teunissen et al., 2015). On the other hand, in the study by Javier, it was found that higher leptin levels were associated with a poorer cognitive function in diabetic patients (Labad et al., 2012). When taking all these studies together into consideration, despite the mechanism that induced the difference of these studies remains to be elucidated, we suggested the most difference between these data was that most of the individuals in those studies showing no protective effect of leptin were obese (measured by BMI). In these individuals, high leptin levels induced leptin resistance indicating a decreased transport of circulating leptin across the blood-brain barrier and therefore a reduction in intracellular signaling downstream (Morrison, 2009), which may partly explain the discrepancy.

WHR, an indicator of visceral fat mass, was found a better predictor than BMI of the risk of T2DM (Cheng et al., 2010). Moreover, the study from Luchsinger et al. exhibited WHR other than BMI associated with increased AD risk (Luchsinger et al., 2012). Similarly, in the current study, WHR rather than BMI was shown to be an independent variable in predicting the risk of MCI in diabetes. Moreover, we also found MOCA or MMSE correlated negatively with WHR and positively with circulating leptin in diabetic women, indicating the protective role of leptin on cognitive function. Additionally, although there was no significant difference, diabetic patients with MCI exhibited higher WHC, WC and HOMA-IR values than those with normal cognitive function, further stratification study showed there was a significant difference in HOMA-IR in women, indicating a decrease in insulin sensitivity. Meanwhile, consistent with the previous study (Hu et al., 2001), our data also demonstrated a facilitated effect of WHR on plasma leptin level. Thus, the current study suggested a complicated relationship among WHR and leptin and cognitive function that alterations in leptin may be involved in the visceral obesity, contributing to cognitive impairment (Davis et al., 2014). Although the mechanism of this relationship is still unclear, here we speculated it may be partly explained by the relatively inadequate leptin secretion following the elevated WHR. Besides, the promotion of inflammation, insulin resistance, reduced adiponectin which is also associated with MCI (Kamogawa et al., 2010; Diniz et al., 2012), and leptin resistance et al. induced by increased visceral fat may also contribute to cognitive deficient (Doumatey et al., 2010; Luchsinger et al., 2012). Of course, these findings should be further confirmed by enlarging the sample size and stratified analysis by WHR. Moreover, an optimum cut-off value of leptin and WHR should be determined to precisely predict the risk of MCI in the future.

Interestingly, in the further stratified analyses by sex, lower leptin levels and FLI and a higher level of sOBR were exhibited in MCI group compared with their counterparts by gender, while the significant difference was only observed in diabetic women. Similar to our result, in a study of women with AD, the leptin levels and FLI were also significantly lower in AD group when compared with the controls (Baranowska-Bik et al., 2015). In fact, the sexual dimorphism of leptin concentration is not uncommon in animal and human studies. It has already been shown that circulating leptin concentration was significantly higher in women with heart failure or diabetes than men (Park and Ahima, 2016; Cundrle et al., 2017). An animal study suggested the brains of male and female rats had differential sensitivity to central leptin, and female rat administered with leptin administration exhibited a long duration of catabolic action (Clegg et al., 2003). Although the detailed mechanism is not yet fully understood, a higher proportion of body fat in women (Cammisotto and Bukowiecki, 2002) and the counter-regulatory effect between leptin and testosterone (Hislop et al., 1999), high level of adiponect in female (Robinson et al., 2011), as well as high estrogen that function as negative regulators of leptin in female amnestic MCI (Xing et al., 2015) may help to explain the significant difference of leptin concentrations in diabetic women.

As noted above, leptin can cross over brain barrier and act through the receptors expressed within the brain in a variety of regions, including the cortex, hypothalamus, the midbrain, and the hindbrain et al. (Arnoldussen et al., 2014; Farr et al., 2015). This specific location of leptin receptors determines its diversity effect on multiple cognitive domains (e.g., study, memory, verbal, spatial, and executive function). In the present study, it was exhibited that higher leptin concentration and FLI were associated with the improved function in most cognitive domains except the speeded executive function (i.e., TMT) in all patients and women group. The similar association between higher leptin and poor performance on TMT was also observed by Gunstad et al. (Vendemiale, 2015). Data from Lieb and colleagues found that higher levels of leptin were associated with higher total cerebral volume and lower temporal horn volume measured with volumetric brain MRI in dementia-free individuals than AD (Baicy et al., 2007). Additionally, a recent study showed that higher leptin levels were associated with smaller volumes of some frontal structure. Both of which may help to explain the negative association between leptin and the performance on executive function (TMTA and TMTB) (Pannacciulli et al., 2007). Subsequently, we suggested circulating leptin may have selective action at least in part on cognitive function due to its receptors expressed in different brain regions. To gain further insight into the effect of leptin on cognition, we need more studies to explore the underlying molecular mechanism in the future.

There were some limitations regarding our study which need to be considered when interpreting the findings. Firstly, our study is a cross-sectional design with small sample size form the same race (Chinese Han) and hard to be further stratified, and most patients are nonobese based on BMI but visceral obesity, especially in women. Secondly, the present study was just focused on leptin level in elderly T2DM with MCI, not covering the whole process of AD. Thirdly, we did not measure the body fat and other parameters (e.g., adiponectin, testosterone, and estrogen), which may also play an important role on leptin and MCI. As such, more longitudinal cohort studies with enlarged sample size, more related parameters, different ages, and different weight status are required to comprehensively expand the understanding of the role of leptin in cognitive function of T2DM.

In conclusion, the present study suggested the selectively neuroprotective role of low sOBR level and high plasma leptin level, which may be a good predictor of MCI (Zeki Al Hazzouri et al., 2013) especially in diabetic women and provided insight that WHR (visceral obesity) may contribute to the development of cognitive impairment in patients with T2DM.

## Conclusion

In summary, the current study showed the control group had a higher plasma leptin level, FLI and lower sOBR level than MCI group. Additionally, high level of leptin, FLI and low level of sOBR were associated with improved cognitive function except for poor performance on executive function in all patients and females. Furthermore, Leptin, sOBR, and WHR were found as independent variables of MCI in all individuals, suggesting low plasma leptin and high sOBR levels may be good predictors of MCI in T2DM patients especially in female individuals. And WHR (i.e., the visceral obesity) contributes to cognitive deficits. Further prospective studies with a large population size including different BMI and WHR status, more parameters and more race/ethnicity groups are needed to confirm these findings.

## Author contributions

SW: contributed to the idea and revised the manuscript. HY: carried out the design, conducted of the study and wrote the manuscript. ST and DG: carried out the data collection. RH and RC: participated in the data analysis. HL and JW: helped data interpretation. All authors read and approved the final manuscript.

### Conflict of interest statement

The authors declare that the research was conducted in the absence of any commercial or financial relationships that could be construed as a potential conflict of interest.
